# *Dirofilaria immitis* and *D. repens* show circadian co-periodicity in naturally co-infected dogs

**DOI:** 10.1186/s13071-017-2055-2

**Published:** 2017-02-28

**Authors:** Angela Monica Ionică, Ioana Adriana Matei, Gianluca D’Amico, Lucia Victoria Bel, Mirabela Oana Dumitrache, David Modrý, Andrei Daniel Mihalca

**Affiliations:** 1 Department of Parasitology and Parasitic Diseases, University of Agricultural Sciences and Veterinary Medicine Cluj-Napoca, 335700 Cluj-Napoca, Romania; 2 Department of Anesthesiology and Surgical Techniques, University of Agricultural Sciences and Veterinary Medicine Cluj-Napoca, 335700 Cluj-Napoca, Romania; 3 Department of Pathology and Parasitology, University of Veterinary and Pharmaceutical Sciences, Brno, Czech Republic; 4 CEITEC-VFU, University of Veterinary and Pharmaceutical Sciences, Brno, Czech Republic; 5 Institute of Parasitology, Biology Centre of the Czech Academy of Sciences, České Budĕjovice, Czech Republic

**Keywords:** Periodicity, Microfilariae, Co-infection, *Dirofilaria immitis*, *Dirofilaria repens*

## Abstract

**Background:**

*Dirofilaria immitis* and *Dirofilaria repens* are mosquito-borne zoonotic filarioids typically infecting dogs, causing a potentially fatal cardiopulmonary disease and dermatological conditions, respectively. The females are larviparous, releasing the larvae (microfilariae) into the bloodstream, which further develop in mosquito vectors. However, microfilaremia greatly fluctuates during a 24-h period. As the sampling time can greatly influence the accuracy of diagnosis, the aim of the present study was to assess the circadian periodicity of *D. immitis* and *D. repens* in naturally co-infected dogs in an endemic area of Romania and to investigate possible differences of periodicity between these two species.

**Methods:**

Overall, four dogs harbouring natural co-infection with *D. immitis* and *D. repens* were selected and sampled every two hours for two consecutive days: two dogs in July 2014 and two in July 2015. At each sampling time, a 0.7 ml blood sample was taken. Modified Knott’s test was performed on 0.5 ml, and the remaining 0.2 ml were used for DNA extraction and molecular amplification, both in single and duplex PCR reactions. Microfilariae of both species were morphologically identified and counted in each collected sample, microfilaremia was calculated, and fluctuation was charted.

**Results:**

The dynamics of microfilaremia showed similar patterns for both *Dirofilaria* species. In all four dogs, *D. immitis* was present at all sampling times, with several peak values of microfilaremia, of which one was common for all dogs (1 am), while minimum counts occurred between 5 and 9 am. Similarly, for *D. repens*, one of the peak values was recorded in all dogs at 1 am, while minimum counts (including zero) occurred at 9 and 11 am. Single species-specific PCR reactions were positive for both *D. immitis* and *D. repens* in all collected samples, while duplex PCR failed to amplify *D. repens* DNA in many cases.

**Conclusions:**

Both *Dirofilaria immitis* and *D. repens* microfilariae are subperiodic, following a similar variation pattern, with peak values of microfilaremia registered during the night in Romania. Duplex PCR fails to identify the infection with *D. repens* in co-infected dogs when the ratio of microfilaremia is in favour of *D. immitis*.

**Electronic supplementary material:**

The online version of this article (doi:10.1186/s13071-017-2055-2) contains supplementary material, which is available to authorized users.

## Background

In Europe, dogs may be infected by various species of filarioids (Spirurida, Onchocercidae). Among these, veterinary attention has been focused mostly on *Dirofilaria immitis*, the heartworm, which poses a great risk to animal health, as it is associated with a potentially fatal cardiopulmonary disease [1]. A second species of zoonotic importance is *D. repens*, which resides in the subcutaneous tissues of the canine host and infection is frequently asymptomatic or associated with a series of dermatological conditions [2, 3]. Both species are regarded as zoonotic agents, but *D. repens* is more commonly reported in humans throughout Europe [4, 5]. The female nematodes are larviparous, releasing blood-circulating microfilariae, which are ingested and later transmitted by several genera of mosquitoes (*Culex*, *Aedes* and *Anopheles*) which act as intermediate host and vector [5]. However, in the case of both species, the number of microfilariae present in the peripheral blood fluctuates during the day, according to several potential factors, including geographic origin, and it is assumed that microfilariae concentrate in the lung vessels during the low peripheral microfilaremia phases [6].

The impact of *Dirofilaria* spp. on animal and human health is recognised throughout Europe, particularly given the recent geographical expansion of both species [7, 8]. However, the level of awareness in non-endemic or newly endemic territories is still low [9]. Furthermore, in many clinical facilities, diagnosis is based solely on the microscopic detection of microfilariae, which may yield false-negative results, due to several factors, including the intermittent presence of microfilariae in the peripheral blood, or identification of only one species in co-infected animals [10, 11]. As the risk of human infection is directly related to the populations of infected dogs, particularly in areas where abundant vector populations are present [12], an accurate diagnosis is crucial for the disease control.

As some areas of Romania are endemic for both *Dirofilaria* species and mixed infections are common [13], while the level of awareness is still low, the necessity of establishing the optimum sampling time to avoid false negativity of diagnostic tests becomes evident. The aims of the present study were to assess the circadian periodicity of *D. immitis* and *D. repens* microfilariae in naturally co-infected dogs in an endemic area of Romania and to investigate possible differences of larval periodicity between the two species.

## Methods

The study was conducted in July 2014 and July 2015 in Chilia Veche (45.421944N, 29.289722E), a rural locality in the Danube Delta region of Romania. Previously, 70 privately owned dogs were tested for the presence of filarioid infection by using modified Knott’s test [14]. Four of the dogs which had *D. immitis* and *D. repens* co-infection were enrolled in the experiment after receiving consent from the owner, as follows: dog 1, a 12 year-old castrated male and dog 2, a 4 year-old male were sampled in July 2014; dog 3, a 5 year-old male and dog 4, a 2 year-old male were sampled in July 2015. All dogs were medium-sized mixed breed, lived exclusively outdoors, never travelled and did not receive any antiparasitic treatments. To avoid excessive stress, 32 mm G20 venous catheters were inserted into the cephalic vein at the beginning of the sampling. The dogs were sampled at a two hours interval for 48 h (= 24 sampling events per dog). At each sampling time, the active/sleeping status of the dog was registered and 0.7 ml of blood was taken into labelled EDTA tubes as follows: 0.5 ml of blood was used for modified Knott’s test (the 2% formalin was added in situ), following the standard proportions and procedures [14]; the remaining 0.2 ml volume was stored at -20 °C until DNA isolation and amplification. In the modified Knott’s test, the volume of the total sediment was measured for each sample, and a 40 μl homogeneous fraction was examined under a light microscope (Olympus BX 61; Olympus, Tokyo, Japan). Microfilariae were morphologically identified [14] and counted. Based on the obtained counts, the total and average microfilaremia were calculated. Considering the total average daily counts for each species as 100% for 24 h, variation charts were generated individually for each species of filarioid, according to sampling time, as a percentage, based on the two days average for the respective sampling time. Genomic DNA was extracted from the remaining 0.2 ml of blood from each sample using a commercial kit (Isolate II Genomic DNA Kit, Bioline, London, UK) according to the manufacturer’s instructions. Two types of PCR reactions targeting fragments of the 12S rDNA and *cox*1 genes were performed both individually for each species and in a duplex for the simultaneous detection of *D. immitis* and *D. repens* DNA, following reaction procedures and protocols described in the literature [15, 16]. In each reaction set, a positive control (DNA extracted from adult nematodes) and a sample with no DNA were included. PCR products were visualised by gel electrophoresis, and their molecular weight was assessed by comparison to a molecular marker (O’GeneRuler™ 100 bp DNA Ladder, Thermo Fisher Scientific Inc., Waltham, MA, USA).

Correlations between microfilaremia values and between the results of the Knott test and duplex PCRs were evaluated using Spearman’s rank correlation test (http://www.socscistatistics.com. Accessed September 2016).

## Results

### Periodicity of microfilaremia

**Fig. 1:**
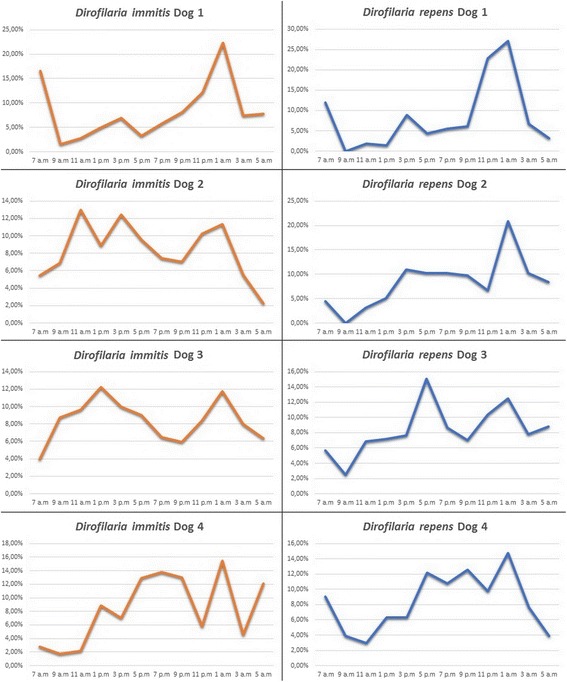
Percentile variation charts, according to sampled dog and filarioid species, considering the total average daily counts as 100%

The average values of microfilaremia for each dog and sampling time are presented in Table 1. Overall, the dynamics of microfilaremia showed similar patterns for both *Dirofilaria* species, in all four dogs (Fig. 1).

**Table 1:** The average values of microfilaremia at each sampling time

	7 am	9 am	11 am	1 pm	3 pm	5 pm	7 pm	9 pm	11 pm	1 am	3 am	5 am
Dog 1 (July 2014)												
*D.i.*	584	56	98	173	245	116	207	288	429	785	261	272
*D.r.*	252	0	38	30	186	91	116	128	479	568	140	68
Dog 2 (July 2014)												
*D.i.*	5,952	7,920	14,190	9,730	13,600	10,440	8,160	7,700	11,220	12,546	6,020	2,426
*D.r.*	372	0	258	420	900	840	840	800	550	1,722	840	694
Dog 3 (July 2015)												
*D.i.*	2,159	4,775	5,277	6,715	5,457	4,920	3,536	3,067	4,586	6,435	4,370	3,480
*D.r.*	217	94	262	272	290	575	330	268	396	478	296	338
Dog 4 (July 2015)												
*D.i.*	855	520	680	2,750	2,170	3,990	4,275	4,025	1,780	4,795	1,395	3,752
*D.r.*	428	182	140	300	298	578	510	595	460	700	360	182

*Abbreviations*: *D.i*. *Dirofilaria immitis* microfilariae/ml, *D.r.* *Dirofilaria repens* microfilariae/ml

*Dirofilaria immitis* microfilariae were present in all dogs at all sampling times, with several recorded peak values. One of the peaks (1 am) was common for all dogs, while others occurred in the morning or afternoon samples. The minimum counts were always recorded in the morning samples, between 5 am and 9 am. Similarly to *D. immitis*, one peak value of *D. repens* microfilaremia (1 am) was common for all animals. Other maximum counts occurred differentially in the afternoon and evening samples. Minimum values, including zero counts (dogs 1 and 2) were recorded between 9 am and 11 am

In two dogs, a strong positive and statistically significant correlation between the dynamics of the microfilariae of the two *Dirofilaria* species was observed (dog 1: *R* = 0.853, *P* = 0.0004; dog 4: *R* = 0.732, *P* = 0.0067). In the other two dogs, a weak correlation with no statistical significance was noted (dog 2: *R* = 0.204, *P* = 0.52; dog 3: *R* = 0.195, *P* = 0.54).

### Molecular assays

Single species-specific PCR reactions were positive both for *D. immitis* and *D. repens* DNA in all collected samples, regardless the target gene. The duplex PCR failed to amplify *D. repens* DNA in certain cases (Additional file 1: Table S1).

In the case of duplex PCR reactions, a statistically significant correlation between the ratio of microfilariae of the two species (*D. immitis*: *D. repens*) and the false negativity for *D. repens* was noted (*R* = 0.592, *P* = 0 for 12S rDNA; *R* = 0.242, *P* = 0.017 for *cox*1).

## Discussion

The periodicity of microfilariae has been first described for *Wuchereria bancrofti* in 1879 [6]. Since then, this phenomenon has been observed for several other species of filarioids, including *Dirofilaria* spp., which seem to have a subperiodic cycle, i.e. microfilariae are always present in the peripheral blood, but in fluctuating concentrations [17]. The present study further suggests the existence of a relatively stable pattern of the circadian periodicity of *D. immitis* and *D. repens* microfilariae in naturally co-infected dogs.

The exact mechanism of periodicity is still unknown, but there are two major theories regarding its occurrence. Some authors consider it in relation to the temporal availability of vectors in the respective geographical area, having a local character [18, 19]. Indeed, reports from various countries seem to support this theory. For *D. immitis*, maximum counts have been recorded at 11 am in Tanzania [20], at 6 pm in England [17], between 7 pm and 9 pm in Korea [21] and between 9 pm and 10 pm in Japan [22]. For *Dirofilaria repens*, maximum counts have been recorded between 10 pm and 3 am in England [17] and throughout the night in Italy [19]. On the other hand, some authors state that the periodic cycle of the microfilariae is in fact oriented to the 24-h habits of the host and varies according to internal factors. For instance, in dogs infected with *D. immitis* that were forced to be active during the night and slept by day, within one week, microfilaremia values shifted, with maximum counts during the day instead of the night [23]. Experimental studies also seem to support the hypothesis that microfilaremia variates according to intrinsic factors of the host [24]. For both *D. immitis* and *D. repens*, microfilaremia rises in anesthetized dogs, in changes of oxygen pressure (in both directions, but more markedly when it decreases) and drops when the animal is hyperventilated [24]. Also, a decrease in the dog’s body temperature was followed by a significant drop in the number of microfilariae of *D. immitis* [23]. During sleep, the body temperature falls, carbon dioxide pressure rises, oxygen pressure decreases, acidity rises, kidneys secrete fewer chlorides, and the adrenals are less active, all of these factors having a potential contribution to the dynamics of microfilaremia [6]. In the present study, in most cases, maximum counts were attained during the night (1 am). This corresponds to the peak biting activity of local mosquito species, such as *Culex pipiens*, which is an efficient vector for both *Dirofilaria* spp. and highly attracted to dogs [25, 26], but also to the sleeping behaviour of the sampled dogs.

To our knowledge, this is the first periodicity study performed on co-infected dogs. The interspecific relationships between *D. immitis* and *D. repens* have been only partially studied, suggesting an inhibition of the development of *D. immitis* in dogs previously infected with *D. repens* [27]. However, our results indicate that once the animal develops a patent co-infection, the microfilariae of the two species display a similar circadian periodicity, probably as a reaction to the same stimuli, with no apparent influences between each other.

In many cases, the duplex PCR reactions failed to amplify the DNA of *D. repens*, while species-specific reactions never yielded false negative results. These results were probably due to a preferential amplification of one DNA template over the other, a frequent phenomenon when using multiplex PCRs [28]. The disproportion of microfilariae between the two species was always in favour of *D. immitis* (Tables 1, 2). Therefore, the use of two different species-specific amplification reactions in regions with an unknown epidemiological situation, or if there is a suspicion of co-infection would be advisable.

Ultimately, knowledge regarding the optimum sampling time would greatly decrease the risk of false negative diagnosis, allowing practitioners to initiate proper therapy, thus increasing the chances of survival and the general welfare of infected animals. Not least, an early initiation of microfilaricidal therapy would greatly reduce the spreading of these parasites.

## Conclusion

Both *Dirofilaria immitis* and *D. repens* microfilariae are subperiodic, following a similar variation pattern, with peak values of microfilaremia registered during the night in Romania. Duplex PCR fails to identify the infection with *D. repens* in co-infected dogs when the ratio of microfilaremia is strongly in favour of *D. immitis*, regardless the sampling time. We recommend the use of species-specific PCR or Knott’s test, performed on evening/night samples.

## Additional file

Additional file 1: Table S1. The proportion of present microfilariae (/ml) at each sampling time and results of duplex PCRs. (XLSX 11 kb)
